# Redefining Chronic Inflammation in Aging and Age-Related Diseases: Proposal of the Senoinflammation Concept

**DOI:** 10.14336/AD.2018.0324

**Published:** 2019-04-01

**Authors:** Hae Young Chung, Dae Hyun Kim, Eun Kyeong Lee, Ki Wung Chung, Sangwoon Chung, Bonggi Lee, Arnold Y. Seo, Jae Heun Chung, Young Suk Jung, Eunok Im, Jaewon Lee, Nam Deuk Kim, Yeon Ja Choi, Dong Soon Im, Byung Pal Yu

**Affiliations:** ^1^Molecular Inflammation Research Center for Aging Intervention (MRCA), Department of Pharmacy, College of Pharmacy, Pusan National University, Busan 609-735, Korea.; ^2^Pathological and Analytical Center, Korea Institute of Toxicology, Daejeon 34114, Korea.; ^3^Department of Internal Medicine, Pulmonary, Allergy, Critical Care & Sleep Medicine, The Ohio State University, Columbus, OH 43210, USA.; ^4^Korean Medicine (KM)-Application Center, Korea Institute of Oriental Medicine (KIOM), Daegu 41062, Republic of Korea.; ^5^Janelia Research Campus, Howard Hughes Medical Institute, Ashburn, VA 20147, USA.; ^6^Department of Internal Medicine, Pusan National University Yangsan Hospital, Yangsan 50612, Korea.; ^7^Department of Biopharmaceutical Engineering, Division of Chemistry and Biotechnology, Dongguk University, Gyeongju 38066, Korea.; ^8^Department of Physiology, The University of Texas Health Science Center at San Antonio, TX 78229, USA.

**Keywords:** chronic inflammation, senoinflammation, aging, senescence-associated secretome, inflammasome, age-related diseases

## Abstract

Age-associated chronic inflammation is characterized by unresolved and uncontrolled inflammation with multivariable low-grade, chronic and systemic responses that exacerbate the aging process and age-related chronic diseases. Currently, there are two major hypotheses related to the involvement of chronic inflammation in the aging process: molecular inflammation of aging and inflammaging. However, neither of these hypotheses satisfactorily addresses age-related chronic inflammation, considering the recent advances that have been made in inflammation research. A more comprehensive view of age-related inflammation, that has a scope beyond the conventional view, is therefore required. In this review, we discuss newly emerging data on multi-phase inflammatory networks and proinflammatory pathways as they relate to aging. We describe the age-related upregulation of nuclear factor (NF)-κB signaling, cytokines/chemokines, endoplasmic reticulum (ER) stress, inflammasome, and lipid accumulation. The later sections of this review present our expanded view of age-related senescent inflammation, a process we term “senoinflammation”, that we propose here as a novel concept. As described in the discussion, senoinflammation provides a schema highlighting the important and ever-increasing roles of proinflammatory senescence-associated secretome, inflammasome, ER stress, TLRs, and microRNAs, which support the senoinflammation concept. It is hoped that this new concept of senoinflammation opens wider and deeper avenues for basic inflammation research and provides new insights into the anti-inflammatory therapeutic strategies targeting the multiple proinflammatory pathways and mediators and mediators that underlie the pathophysiological aging process.

The inflammatory process is an essential immunological defense system in living organisms that has evolved to enhance species survival. Short-term, acute inflammation is a first-line defense mechanism that acts against harmful agents, such as pathogens, toxins, or allergens. Under normal conditions, the tightly coordinated actions of various defense components including immune cells, endogenous anti-inflammatory agents, and tissue remodeling processes enable the resolution of acute inflammation by facilitating the elimination of pathogens, infected cells, and repair to damaged tissues to restore body homeostasis [1].

However, when this intricate acute inflammatory response fails to resolve and persists, more defense components are mobilized to create a long-term unresolved immune response known as chronic inflammation. Chronic inflammation, which typically manifests itself in a low-grade manner for a prolonged period, involves macrophage- and lymphocyte-accumulated leukocytes [2], and various other cellular components. It is important to recognize that this chronic inflammation is causally associated with changes in the cellular redox state and cell death signaling pathways [3].

One of the major changes that occur during aging is the dysregulation of the immune response, leading to a chronic systemic inflammatory state. Among the dysregulated proinflammatory mediators, cytokines and chemokines are major culprits in the development of chronic inflammation and the immunosenescence process.

For instance, interleukin (IL)-6, tumor necrosis factor (TNF)-α, and their receptors, are upregulated in aged tissues and cells [4]. Elevated levels of chemokines and C-reactive protein (CRP) have been found to be involved in age-related pathogenesis [5]. We have previously reported that several key intra- or inter-cellular signaling pathways are closely associated with age-related chronic inflammatory changes during aging [3,6-9].

In the aging literature, there are currently two major hypotheses related to age-related inflammation: inflammaging [10,11] and molecular inflammation [3,12-15]. These two are complementary to each other to a large extent but differ in their focus on age-related inflammatory phenomena. However, recent advances in the inflammation field have made it abundantly clear that age-related chronic inflammation needs to be comprehensively defined at the molecular, cellular, and systemic levels. Because chronic inflammation is so widely and deeply involved in many age-related chronic disorders such as atherosclerosis, diabetes, obesity, sarcopenia, and Alzheimer’s disease [15], it is necessary to establish a new pathophysiological basis for chronic inflammation in relation to the aging process.

**Figure 1.**
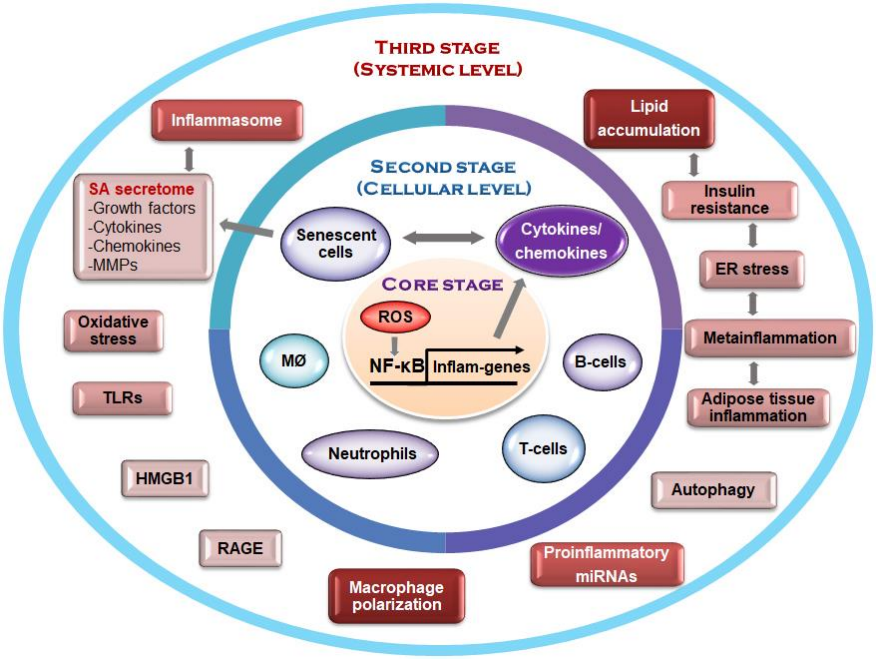
Schematic representation of the senoinflammation concept. MMP, matrix metalloproteinase; Infla-genes, proinflammatory genes; ER, endoplasmic reticulum; TLRs, Toll-like receptors; HMGB1, high-mobility group box 1; RAGE, receptor for advanced glycation end product.

This review summarizes the current knowledge in the field of age-related inflammation. We further discuss the proinflammatory pathways involved in regulating the immunosenescence process and age-related chronic inflammation. We present a new concept with an expanded view of the overall picture of age-related chronic inflammation, which we call senescent inflammation (in short, “senoinflammation”). The salient feature of this concept is to incorporate many proinflammatory mechanisms that have not been previously considered to be important in age-related chronic inflammation.

## Current understanding of age-related inflammatory processes

### Molecular level

The nuclear factor (NF)-κB signaling pathway has been recognized as the most important key process underlying inflammation. Several studies, including ours, have reported that age-related NF-κB signaling upregulates the expression of the proinflammatory genes, TNF-α/β, ILs (IL-1β, IL-2, and IL-6), chemokines (IL-8; regulated on activation, normal T cell expressed and secreted [RANTES]), and adhesion molecules (AMs) [7-16], as shown in Figure 1. Furthermore, NF-κB-mediated upregulation of proinflammatory mediators such as CRP, IL-6, and TNF-α has been shown to be closely associated with various age-related chronic pathophysiological conditions [3].

The important role of NF-κB in maintaining immune responses during age-related inflammation involves activation of proinflammatory cells, leading to the increased expression of various cytokines and chemokines. Adler et al. [17] used motif mapping of the promoters of genes upregulated with aging and concluded that NF-κB is the transcriptional factor most closely associated with aging [17]. In addition, chronic activation of NF-κB has been detected in tissues including the skin, kidney, cardiac muscle, and brain (cerebellum and hypothalamus) [7,18-21]. Several aging studies on NF-κB, and its related signaling have provided important molecular insights into the altered cellular signaling systems that underlie chronic inflammation during aging. These signaling pathways include the insulin and insulin-like growth factor (IGF) pathways, 5'-AMP-activated protein kinase (AMPK)-mechanistic target of rapamycin (mTOR) pathway, Forkhead box O (FOXO) families, sirtuin (SIRT), and p53-related pathways [22].

Data from our laboratory have provided a molecular insight into how chronic stimulation of NF-κB activates inflammatory processes [23, 24]. Our studies have documented that age-associated NF-κB activation is exquisitely sensitive to redox state and oxidative stress, and subsequently leads to increased mitogen-activated protein kinase (MAPK)/inhibitor of NF-κB (IκB)-IκB kinase (IKK) signaling [25]. One interesting recent study has reported the activation of NF-κB occurs in the hypothalamus during aging [20]. The authors of this paper suggested that chronic activation of NF-κB signaling causes hypothalamic inflammation, which then affects whole-body metabolism, in particular, the endocrine regulation of glucose and lipid metabolism [20]. These findings render support for the possibility that dysregulated local tissue inflammation affects the systemic metabolic responses of the whole organism. These new revelations indicate that increased cellular inflammatory signaling pathways and tissue inflammation can propagate to systemic inflammation during the aging process.

### Cellular level

The most noticeable cellular inflammatory changes in age-related chronic conditions are associated with macrophages. The well-known biological activities of macrophages include M1/M2 polarization, phagocytic activity, Toll-like receptor (TLR) signaling, and wound repair [26]. A decline in the expression of macrophage cell surface receptors, such as the major histocompatibility class (MHC)-II protein, has been reported in both aged mice and humans [27,28]. In addition, interferon (IFN)-γ-induced antigen-presenting capacity has been shown to be decreased by 50% in aged mice. Moreover, TLR signaling, M1/M2 polarization, and NF-κB signaling also differ in aged macrophages compared with young macrophages [28, 29]. Data in the literature clearly indicate that there is a substantial dysregulation of macrophage activities during aging, in particular, their ability to produce various pro-inflammatory cytokines. This latter effect can be attributed to a redox imbalance in these dysregulated aging macrophages.

Aberrant increases in macrophage migration and infiltration into various tissues has been noted to be a common occurrence, as evidenced by the massive accumulation of macrophages in adipose tissue during aging [30]. In fact, it has been reported that almost all tissues including the liver, muscle, adipose, brain, kidney, and heart showed increased macrophage infiltration with aging [31-33]. Such a chronic increase in macrophage infiltration into these various tissues is likely to trigger the proinflammatory process at the tissue level.

Increases in innate immune macrophages are accompanied by increases in neutrophils, as well as adaptive immune cells, such as natural killer (NK), B, and T cells. Although the specific types and functions of these cells in aged tissue differ according to the animal models studied and the tissue type, it is evident that increased immune cell infiltration contributes to enhanced chronic inflammation during aging [34].

**Table 1:** Proinflammatory SA secretome in senescent cells, aged tissues, and human tissues.

SASP Factors	Senescent cells	Aged tissues	Human tissues
**Cytokines, chemokines, and regulators**			
IL-1α	↑↑↑	-	
IL-1β	↑↑	↑↑	
IL-6	↑↑↑	↑↑↑	
IL-7	↑↑↑	↑↑	↑
IL-13	↑↑	-	↑
IL1R1	↑	↑	
IL11	↑	↑↑↑	
IL15	↑	-	
IL6R	↑	↑↑	
IL27Rα	↑	-	
IL2RA	↑	↑↑↑	↑
IL-8	↑↑↑	-	
GRO-α (CXCL1)	↑↑↑	-	↑
GRO-β (CXCL2)	↑↑↑	-	↑
GRO-γ (CXCL3)	↑↑↑	-	↑
MCP-1 (CCL2)	↑↑↑	↑↑↑	
MCP-2	↑↑↑	-	
MIP-1α (CCL3)	↑↑↑	-	↑
MIP-3α	↑↑	↑↑↑	
TNF-α	-	↑	↑
TNF-β	-	↑↑	↑
sTNFRI(TNFRSF1B)		↑↑	↑
OPG(TNFRSF11B)		↑*	↑
**Other proinflammatory factors**			
MMP1	↑↑↑	-	
MMP3	↑↑↑	↑↑	
MMP10	↑↑↑	-	
MMP12	↑↑	↑↑↑	
MMP13	↑↑	-	
MMP14	↑↑	-	↑
TIMP1		↑↑↑	↑
iNOS	-	↑↑↑	↑
IGFBP2	↑	-	↑
IGFBP3	↑	↑*	↑
IGFBP6	↑	↑	↑
HGF	↑	↑	↑
EGFR	↑	↑	↑
FAS	↑	↑	↑
Reference	144-146	24, 125	TCGA data base

Recent research on adipose tissue changes during aging has provided considerable insights into its role in age-associated chronic inflammation [35]. Adipose tissue, the largest organ in some organisms, is a major risk factor for the development of the metabolic syndrome during aging because high levels of total and visceral fat are tightly associated with high proinflammatory cytokine levels [36]. Macrophage infiltration into adipose tissue, which is responsible for the local and systemic production of proinflammatory cytokines, may, therefore, be a major cause of the chronic inflammation and metabolic problems in the aged subject [37]. Although macrophage infiltration partly explains how proinflammatory mediators are chronically upregulated with aging, the mechanisms that drive this increased infiltration during aging have not yet been clearly identified.

With increasing age, senescent macrophages (M2-like phenotype) exhibit decreased proinflammatory cytokine secretion, impaired phagocytosis and chemotaxis, and proliferation [38-40]. Macrophages are pre-programmed to clear senescent cells that produce a senescence-associated (SA) secretome, commonly referred to as the senescence-associated secretory phenotype (SASP) (Table 1). It has been suggested that insufficient clearance of senescent cells by senescent macrophages prolongs the inflammatory processes, i.e., chronic inflammation, because the SA secretome contributes to tissue inflammation [41]. Section *SA secrotome* includes a further discussion on the role of the SA secretome in aggravating chronic inflammation.

### Systemic level

Current evidence strongly indicates that increased systemic inflammation is closely associated with aging and age-related chronic diseases [42,43]. As mentioned earlier, this age-related systemic inflammation is distinctly and functionally different from acute inflammation due to the sustained high levels of proinflammatory mediators that are present. It is now well recognized that adipose tissue is one of the major sites involved in systemic inflammation. Increased macrophage infiltration into the adipose tissue environment provides a new paradigm for resident macrophage, leading to the production of various inflammatory cytokines that induce not only adipose tissue inflammation but also propagate systemic inflammation [37]. Because aging is accompanied by increased adiposity, macrophage infiltration further aggravates age-related systemic inflammation [44]. Indeed, both epidemiological and experimental evidence indicates that a state of low-grade, chronic, subclinical inflammation persists in elderly populations of aged animals; these observational and experimental data have served as the basis for the inflammaging hypothesis [45, 46]. More interestingly, a recent longitudinal study of Japanese semi-supercentenarians revealed that inflammation, not telomere length, predicts successful aging at an extremely advanced age [47]. The authors concluded that chronic systemic inflammation had a greater effect on mortality and loss of cognitive function in these centenarians, showing chronic inflammation as an important malleable factor in the aging process [47].

Michaud et al. have shown that two to four-fold elevations in the circulating levels of inflammatory cytokines, such as TNF-α, IL-6, and IL-1β, CRP and serum amyloid A, are typically observed in the elderly or aged animal compared to the young [48]. These observations are noteworthy because the levels of cytokines increase even in healthy individuals in the absence of acute infection or diseases [49]. Age-associated increases in systemic inflammation are associated with, and predictive of, many aging phenotypes. For example, aberrantly increased inflammation is commonly associated with tissue dysfunction, metabolic syndrome, immune dysfunction, and neuronal problems [48].

Interestingly, evidence shows that proinflammatory mediators can interact with one another and the magnitude of this interaction increases as the level of proinflammatory mediators increases. For example, TNF-α plays an important role in the production of IL-6 by activating several pathways, and IL-6 is a major factor in the elevation of CRP levels found in older adults [50]. Furthermore, as discussed in section *RAGE*, advanced glycation end-products (AGE) and high-mobility group box 1 (HMGB1), which have not previously been considered to be inflammatory mediators, are now thought of as being diverse systemic inflammatory mediators of aging [51,52]. Although systemic inflammation has been proposed to be a major factor associated with increased morbidity and mortality during aging, the precise molecular mechanism remains to be determined. Therefore, the development of a successful anti-aging therapy, aimed at suppressing chronic systemic inflammation, will require a detailed understanding of this mechanism as well as the identification of suitable molecular targets [43].

### Inflammation as a major underlying risk factor for chronic diseases

A chronic inflammatory state is commonly observed in aging and various age-related chronic diseases. The involvement of inflammation in various chronic diseases has been discussed in our previous review, as well as by others [42,53,54], and we will now briefly describe the molecular details.

Metabolic disorders including obesity, insulin resistance, type 2 diabetes, and fatty liver disease are casually associated with inflammation. Metabolically active tissues such as adipose tissue, liver, muscle, and pancreas are common sites of inflammation in aging [55-57]. Proinflammatory factors, acting in either autocrine or paracrine ways, interrupt normal tissue function, as seen for example in insulin resistance. Among metabolic tissues, chronic inflammation in adipose tissue has been well documented and is known to contribute to increased systemic inflammation, indicating an important link between obesity and its pathophysiological consequences [58]. Fatty liver diseases are also associated with inflammation [59].

The inflammatory process also plays an important role in the pathogenesis of atherosclerosis [60]. Leukocyte recruitment and an increase in proinflammatory cytokines are characteristic of the early stages of atherogenesis. Vessel wall cell-derived cytokines also participate in the innate immune response in atherosclerosis. Inflammatory pathways further promote the thrombotic complications of atherosclerosis responsible for stroke and myocardial infarction [61]. Libby et al. have reported that targeting the infiltration of immune cells, or proinflammatory mediators, causes a reduction in atherosclerosis in animal models, as well as in clinical studies [62]. A recent review article by Biasucci et al., focusing on inflammation as the underlying cause of cardiac dysfunction, has highlighted the intricate involvement of oxidative stress, autophagy, damage-associated molecular patterns (DAMPs), TLR4 signaling, and the contribution of the NLRP3 inflammasome [63].

Recent evidence has also shown that chronic inflammation is an underlying cause of age-related neurodegenerative diseases. For example, several proinflammatory cytokines have been implicated in dementia and cognitive decline [64]. Recent evidence strongly indicates the potentially harmful role of microglia (brain-specific macrophages) in the development of dementia, highlighting the importance of the immune-inflammation link [65]. It is well known that microglial activation signifies a primary inflammatory state and causes secondary leukocyte invasion, which amplifies inflammation [65]. Moreover, brain astrocytes and oligodendrocytes also participate in the inflammatory process by producing or responding to, proinflammatory mediators [66].

Similar to other inflammatory diseases, dementia and Alzheimer’s diseases are also associated with the aberrant expression of inflammatory mediators such as complement factors, cytokines, Toll-like receptors (TLRs) and other pattern recognition receptors, lipid metabolites derived from cyclooxygenase and lipoxygenase, and other soluble signaling proteins [66]. Another crucial disease where chronic inflammation is involved in cancer, which has been of tremendous interest after the discovery that inflammation plays an important role in tumor progression [67]. It is now clear that inflammatory cells are indispensable participants in neoplastic formation, cancer cell proliferation, survival, and migration [68]. With respect to tumor progression, it is important to note that tumor cells also share common signaling molecules with the innate immune system, including cytokines/chemokines and cell adhesion molecules [69].

Current evidence strongly suggests that NF-κB, the central core inflammatory mediator, is a key transcriptional factor in the initiation and progression of cancer [70]. Activated NF-κB stimulates both the production of proinflammatory mediators and inhibits cancer cell death. Moreover, NF-κB interacts with other transcriptional factors such as signal transducer and activator of transcription 3 (STAT3) and p53, which are also implicated in cancer, to facilitate cancer initiation and progression [71,72]. Crosstalk can also occur at the level of upstream signaling components, as opposed to at transcriptional level. Glycogen synthase kinase 3 (GSK3)-β, MAPK, or protein kinase B (PKB), which have all been implicated in cancer, also modulate NF-κB transcriptional activity [70]. These lines of evidence support the involvement of the inflammation process, via NF-κB signaling, in the induction and progression of cancer.

## How is age-related inflammation viewed at present?

### Acute and chronic inflammation in aging

As mentioned earlier, the inflammation progresses in two stages: a short-term resolvable inflammatory state and a long-term unresolved chronic inflammatory state. The most powerful players in acute inflammation are tissue-resident macrophages, neutrophils, and mast cells since they act as the first line of defense. Pattern recognition receptors on these cells initially recognize harmful stimuli such as pathogen-associated molecular patterns (PAMPs), damage associated molecular patterns (DAMPs), or both. The most well-known receptors involved in these recognition processes are TLRs and NOD-like receptors (NLRs). Following recognition, signal transduction pathways activate transcription factors such as NF-κB and activator protein (AP)1 [61]. These transcription factors induce the expression of genes that initiate the production of several inflammatory factors including cytokines, chemokines, eicosanoids, and other active proinflammatory molecules. Further activation of other immune cells also occurs in acute inflammation. Ultimately, a successful acute inflammatory response eliminates the cause of the inflammation thereby maintaining the homeostasis of the individual [73].

During the resolution process, newly uncovered anti-inflammatory players including lipoxins, resolvins, protectins, and other eicosanoids are now known to play key roles [73]. A recent report by Arnardottir et al. [74] has demonstrated that aged mice show a delayed resolution of acute inflammation because of reduced levels of these specialized pro-resolving lipid mediators.[61].

In contrast, un-resolved, low-grade inflammation follows a different path from acute inflammation. Further recruitment of macrophages, along with the appearance of T cells, replaces the initial neutrophil population in the acute phase of inflammation. These secondary immune cells attempt to eliminate the cause of the inflammation. However, they usually fail to resolve the initial inflammation, leading to a chronic inflammatory state with the formation of ectopic lymphoid-like structures such as granulomas [75]. Although the exact mechanisms and inflammation processes differ in various chronic inflammatory states, the consequences are similarly associated with pathological conditions such as autoimmune diseases, fibrosis-related diseases, cancer, and other degenerative diseases.

### Brief descriptions of the two hypotheses of age-related inflammation

In the aging literature, two major hypotheses concerning the involvement of chronic inflammation in aging have been proposed: molecular inflammation [3,12-14] and inflammaging [10,11].

#### 1) Molecular inflammation

This hypothesis was first proposed by our laboratory in 2002, based on molecular changes in inflammation-related transcription factors and in the expression levels of their target genes. The hypothesis states that these changes are the mechanism underlying the aging process and age-related diseases [14,15]. The validity of this hypothesis stems from the extreme sensitivity of the transcriptional factor NF-κB to oxidative stress and to changes in redox balance [3]. Incessant oxidative stress and compromised antioxidant defense systems during aging are blamed for increased reactive species (RS), including reactive oxygen species (ROS), reactive nitrogen species (RNS), and reactive lipid aldehydes [3]. Although young organisms have a well-functioning antioxidant system to maintain redox balance, the age-related decline in the anti-oxidant defense system fails to maintain redox homeostasis, leading to the activation of various proinflammatory signaling pathways.

Altered redox signaling pathways can further increase various redox-sensitive transcription factors, in addition to NF-κB and AP1, during aging. Cellular redox signaling generally activates protein tyrosine kinases/protein tyrosine phosphatases (PTKs/PTPs) located near the plasma membrane, which further activate serine/threonine kinases/phosphatases [25]. This imbalance in PTKs/PTPs during oxidative stress further activates various downstream kinase such as the NF-κB-inducing kinase (NIK)/IKK and MAPK. This kinase further activates age-related NF-κB activation. Consequently, the gene expression of proinflammatory cytokines (e.g., TNFα, IL-1β, and IL-6) as well as cyclooxygenase-2 (COX-2), lipoxygenase (LOX), inducible nitric oxide synthase (iNOS), and AMs (vascular cell AM 1 [VCAM-1], intercellular AM 1 [ICAM-1], and E-selectin) are all upregulated through NF-κB activation during the aging process [16,25,76]. It is worth emphasizing here that NF-κB act as a master transcription factor for many proinflammatory genes, pathways, and other mediators, including the SA secretome (See Fig. 1).

#### 2) Inflammaging

An age-related phenomenon of a progressive increase in proinflammatory status was originally termed “inflammaging” by C. Franceschi and his group in 2000. Inflammaging is manifested by increased pro-inflammatory cytokines that are commonly observed during aging [77]. This novel concept states that activation of the aged innate immune system leads to a dysregulation in inflammation that impairs the ability to initiate an efficient innate and adaptive immune program in responses to antigens or environmental stimuli (e.g., ROS) [78]. Various aging studies have produced data in support of inflammaging in aged mice or human subjects exhibiting elevated steady-state levels of inflammatory cytokines, acute phase proteins, clotting factors, stress hormones, and redox stress [10,79]. According to this hypothesis, these alterations in the immune system contribute to the development of overt organ-specific inflammatory diseases such as atherosclerosis, Alzheimer’s disease, and diabetes [80]. Although the inflammaging theory has served well in describing the phenomenon of age-related inflammation that modulates the course of aging and age-related diseases, the detailed mechanisms behind inflammaging are sparse.

#### 3) More players participate in chronic inflammation

Newly emerging data has revealed that age-related chronic inflammation is much more widely and heavily involved in many cellular activities than previously thought. One example is that decreased autophagic function is implicated in age-related inflammation [81]. Autophagy plays an essential role in the removal of dysfunctional intracellular proteins by lysosomal degradation. Recently, it has been reported that the autophagic response is diminished in lipopolysaccharide (LPS)-treated aged rats and that lipid metabolism is impaired during sepsis, indicating that the autophagic response is important in regulating lipid metabolism after endotoxin challenge [82].

The autophagic response declines with age and this impairment potentially leads to the activation of inflammasomes. Inflammasomes, intracellular sensors for detecting pathogenic agents and sterile stress, activate proinflammatory cytokines as a consequence of tissue injury or necrosis [81]; thus, the impaired autophagic function associated with age likely results in chronic inflammatory responses through a defective regulation of the cellular inflammasomes system (See more on inflammasomes in Section *Inflammasome)*.

As mentioned earlier, during aging, adipose tissue mass increases in various tissues such as the liver, bone, and muscle. This age-dependent increase in tissue adiposity can locally and systemically influence inflammatory responses by increasing the secretion of adipokines [83]. These adipokines, which are adipose-derived cytokines and chemokines, lead to immune cell recruitment to the adipose tissue and induce the production of a number of proinflammatory cytokines. Consequently, the increased adiposity of various tissues seen during aging contributes to an increase in the proinflammatory environment, partly via increased adipokine production.

It is important noting that these age-related changes in adiposity, autophagy, and inflammasome that exacerbate age-related chronic systemic inflammation are not considered in the current, conventional, view of chronic inflammation.

Other important participants in the chronic inflammation field are new mediators of inflammatory signaling pathways. These factors include molecules such as non-coding microRNAs (miRNAs), mitochondrial DNA, and N-glycosylated proteins that are found in the circulatory system and can influence inflammatory state during the aging process [84]. A more detailed description of these aspects is described in the following section.

## Age-related cellular factors and processes exacerbating age-related chronic inflammation

### ER stress

The endoplasmic reticulum (ER) is a cellular organelle that plays a central role in maintaining proteostasis because of its involvement in protein synthesis, folding, maturation, quality control, distribution, and degradation [85]. With respect to insulin signaling, the ER has been found to be associated with insulin resistance [86]. ER stress induces serine phosphorylation of insulin receptor substrate 1 (IRS-1) via the c-Jun N-terminal kinase (JNK) pathway, which then inhibits insulin responses in cultured liver cells [55,87] enhancing lipogenesis, affecting hepatic steatosis, and influencing insulin resistance [88]. Thus, the ER may be a proximal site that senses over-nutrition and translates it into metabolic and age-related inflammatory responses.

Lipids play a wide variety of roles under pathophysiological conditions. A wide-spread abnormal accumulation of lipids in adipose tissue, as well as ectopic sites such as the liver and muscle, during aging, provides a great opportunity for ER stress and the activation of proinflammatory genes in numerous tissues. Furthermore, ER stress has been demonstrated to activate JNK, as well as IKK, by increasing IRE1, thereby inducing NF-κB activation [89, 90]. Increased JNK and NF-κB signaling then induce insulin resistance and the expression of proinflammatory cytokines [91].

The notable association between the metabolic syndrome and the aging process indicates that inflammation underlies the onset and progression of metabolic syndrome [92]. Furthermore, it has been established that insulin resistance is potentiated and induced by the proinflammatory cytokine TNF-α, as well as other cytokines that are upregulated during aging [93].

Many studies suggest that ER stress and insulin resistance are associated with lipid accumulation, leading to an exacerbation of inflammation and age-associated chronic inflammation. Prolonged ER stress leads to both inflammation and cell death [94], and recent studies have shown that ER stress-induced inflammation and cell death are mediated by NOD-like receptor (NLR) family pyrin domain containing 3 (NLRP3) inflammasome activation [95]. NLRP3 activates caspase-1, which then processes pro-IL-1β to the mature, secreted IL-1β form [96,97]. Zhang and Kaufman [94] have reported that suppressing ER stress-associated NLRP3 inflammasome activation might be an effective therapeutic strategy for blocking the vicious cycle of inflammation and adipose dysfunction in age-related diseases.

### Inflammasome

The NLRP3 inflammasome is an intracellular multiprotein complex that can recognize pathogen- and DAMP [98]. NLRP3 activation leads to the production and secretion of IL-1β as well as IL-18 [98]. The NLRP3 inflammasome has been shown to play a central role in obesity, insulin resistance, and inflammation [99,100]. Activation of the well-studied NLRP3 inflammasome is achieved through a diverse array of molecules that can be sensed by cell surface receptors and this activation is thought to participate in aging-related inflammatory processes. Aged NLRP3-deficient mice have a significant increase in naive T cells along with a reduction in effector-memory cells. These findings suggest that the NLRP3 inflammasome causes thymic involution by sensing the age-associated increase in intrathymic “lipotoxic danger signals,” and that dampening of NLRP3 inflammasome activation may enhance naive T cell production by the thymus. The NLRP3 inflammasome, therefore, controls the aging of the thymus and lead to immunosenescence.

Hanouna *et al.* have recently reported that suppression of the NLRP3 inflammasome extends lifespan in mice by attenuating age-related degenerative changes, including cognitive decline [101]. Based on their findings, Youm et al. [102] have proposed that the suppression of aberrant NLRP3 activity during aging may attenuate age-related diseases by reducing chronic inflammation. In addition, aged mice failed to show upregulation of TLR1, TLR2, NOD2, NLRP3, and IL-1β in response to colonization. Baseline inflammation in aged mice, along with a failure to upregulated innate response genes, could impede the signaling that promotes monocyte/macrophage influx and, thus, explains the delayed clearance [103].

Youm *et al*. have also shown that pharmacological NLRP3 inflammasome blockers, which specifically target the thymus, may delay immunosenescence, maintain a diverse T cell repertoire, and enhance immune-reconstitution in elderly patients [102]. Furthermore, aged mice developed lung fibrosis and exhibited increased morbidity and mortality after bleomycin-induced lung injury in NLRP3 activation. Both bone marrow-derived macrophages and alveolar macrophages from aged mice display higher levels of NLRP3 inflammasome activation and caspase-1-dependent IL-1β and IL-18 production than the same macrophages from younger mice [104]. Furthermore, these effects are associated with altered mitochondrial function and increased ROS production [104].

It is worth mentioning that we have recently observed increased hepatic inflammasomes during aging (unpublished data). Age-related activation of the inflammasomes, therefore, exacerbates immunosenescence and inflammation in the aging process, leading to age-related chronic inflammation.

### HMGB1 and receptor for AGE (RAGE)

Damage-associated molecular patterns (DAMPs) are molecules released by stressed cells undergoing necrosis that act as endogenous danger signals to promote the inflammatory response [105]. This response by DAMPs is also called “sterile inflammation” because it is initiated in response to inflammatory insults such as trauma or ischemia in the absence of pathogen infection [106]. DAMPs include the chromatin-associated protein HMGB1, heat shock proteins (HSPs), cytokines including IL-1β and IL-33, DNA/RNA, S100 molecules, purine metabolites, and hyaluronan fragments. They are expressed in different cell types and function in normal cellular homeostasis, but increased serum levels of DAMPs have been associated with many inflammatory diseases including sepsis, rheumatoid arthritis, diabetic nephropathy, atherosclerosis, and neurological diseases [107].

In this review, HMGB1 and the receptor for advanced glycation end product (RAGE) are discussed in relation to age-related inflammation. HMGB1 is a member of the non-histone nuclear protein family and is a highly conserved gene that is expressed in all eukaryotic cells. Under normal conditions, HMGB1 binds to the minor groove of DNA and bends it to facilitate gene transcription, but under stressed conditions such as injury or infection, HMGB1 is released and promotes inflammatory responses [108]. Elevated HMGB1 levels have been reported in aging and various inflammatory diseases such as sepsis, rheumatoid arthritis, and cancer [109,110].

The release of HMGB1 can be triggered by different inflammatory mediators such as LPS, IL-1β, and IFN-α and it induces the NF-κB or JAK/STAT signaling pathways, thereby potentiating inflammatory responses [111,112]. Although the signaling pathways elicited by HMGB1 are not fully defined, HMGB1 has been reported to trigger the activation of key signaling pathways by binding to RAGE, TLR-2, TLR-4, and TLR-9 [113] (there is a further discussion of TLRs in part d below).

RAGE was the first identified receptor for HMGB1 [114] and is a member of the immunoglobulin superfamily and is expressed on mononuclear phagocytes, vascular smooth cells, neurons, and a variety of tumor cells [115]. RAGE, as a multi-ligand receptor, interacts with HMGB1, as well as various other ligands such as AGE, S100 proteins, and β-amyloid [115]. HMGB1-induced RAGE signaling activates MAPKs, phosphoinositide 3-kinase (PI3K)/Akt, JAK/STAT, Src family kinases, and NF-κB and has been implicated in various chronic inflammatory diseases [116]. Furthermore, HMGB1/RAGE induces IL-17 expression, which aggravates inflammation in the peripheral blood cells of patients with hepatitis B [117]. An elevated HMGB1 expression has been identified in smokers with chronic obstructive pulmonary disease (COPD) [118]. In addition, released HMGB1 not only induces p53 activity and inflammation in senescent fibroblasts [119], but is also involved in proinflammatory responses in the aged kidney [109] and brain [52].

### TLR signaling changes

Toll-like receptors (TLRs) are a family of pattern recognition receptors involved in initiating innate immune system response to microbes and tissue damage [120]. TLRs are widely expressed in the cells of many tissues including epithelial, endothelial, dendritic, monocytes/macrophages, and B- and T-cells [121]. The human and the mouse TLR family contain ten and thirteen members, respectively [122].

Accumulating evidence indicates that aging and chronic inflammations are closely associated with increased TLR expression. TLR5 expression and TLR5-induced production of IL-8 were found to be higher in monocytes from older individuals than in those from younger individuals [123]. Elevated TLR4 expression and pro-inflammatory signaling have been observed in the muscle of older individuals, and these alterations were associated with decreased insulin sensitivity and muscle loss [124]. Our recent results show that there is an increase in expression of TLR7 and proinflammatory cytokines in aged rat kidneys [125].

Several studies have reported that TLRs are associated with age-related inflammatory diseases [124]. Because of the tight link between TLRs and inflammatory diseases, TLR4 is extensively involved in renal fibrosis and chronic kidney disease progression [126] and the expression of TLR2 and TLR4 mediates ischemia/reperfusion injury [127]. In addition, TLR7 and TLR9 contribute to the development of glomerulonephritis in systemic lupus erythematosus [128]. Therefore, TLRs may provide mechanistic support for a close link between chronic inflammation and the aging process.

### Non-coding miRNAs

Non-coding miRNAs comprise a highly conserved family of small RNAs (18-22 bp in length) that generally act as negative post-transcriptional regulators of gene expression. They are predicted to regulate the expression of more than 50% of human protein-coding genes acting through mRNA destabilization and/or translational repression. The miRNAs control a wide array of biological processes such as cell differentiation, proliferation, and apoptosis [129].

New emerging data has shown that several miRNAs are involved in regulating inflammation; their prototypes are miR-155, miR-21, and miR-146a [130], often referred to as inflamma-miRs. These miRNAs have also been implicated in aging and age-related inflammatory disease. In a cohort study, circulating levels of miR-21 in the plasma of aged subjects and animals increased with age and there were positive correlations between miR-21 levels and two important aging biomarkers, namely CRP and fibrinogen [131]. In addition, centenarians had lower miR-21 levels than healthy 80-year-old subjects, suggesting that low levels of miR-21 could be a useful biomarker of longevity [132]. Interestingly, miR-155 expression in the peripheral blood of older women was higher than in young adult women [133]. Furthermore, miR-146a plays an important role in the resolution of inflammation but shows altered expression in the plasma from patients with cardiovascular disease [134] and Alzheimer’s diseases [135].

Several miRNAs have been shown to modulate specific signaling pathways including the NF-κB, mTOR, SIRT, transforming growth factor (TGF)-β, and Wnt signaling pathways that are thought to be related to inflammation, cellular senescence, and age-related diseases [136]. In aging tissues aging as well as cellular senescence, dysregulated miRNAs have been shown to be involved in the insulin signaling pathway (e.g., let-7), the DNA damage response (e.g., miR-34), mitochondrial function (e.g., miR-146a), and cell death (e.g., miR-30e) [137,138]. Thus, altered expression of the miRNAs targeting these pathways may contribute to dysregulation of the inflammatory/anti-inflammatory balance, promoting aging. Moreover, it has recently been observed that miRNAs act as TLR ligands, inducing NF-κB signal activation and IL secretion, thus triggering a proinflammatory response [139]. For example, Bernard et al. [140] have shown that damaged RNAs released from ultraviolet B (UVB)-exposed keratinocytes activate TLR3 on intact keratinocytes, which initiates the cutaneous inflammation associated with sunburn. In addition, Chen et al. [141] have reported that RNA released from necrotic cells after ischemia-reperfusion (I/R) contributes to ischemic myocardial injury through TLR3-Trif signaling and that RNase treatment reduced inflammation, apoptosis, and infarction during I/R. Therefore, several age-related miRNAs play important roles in regulating chronic inflammation and the aging process.

### Exacerbation of age-related chronic inflammation by senescent-associated (SA) secretome

Cellular senescence has been considered by many as a root of the aging process and age-related diseases. A recent renewed interest in cellular senescence has arisen due to the recognition that senescent cells have harmful effects on the host organism. The removal of senescent cells, identified using the p16 Ink4a-biomarker, by injecting a senolytic agent twice weekly, starting at one year of age, extended the median lifespan of mice by approximately 30% [142]. It is becoming clear that senescent cells can have seriously deleterious effects, interfering with various normal cellular functions and promoting the pathological process, including chronic inflammation, deterioration of the immune system, and age-related tumorigenesis.

**Table 2:** Comparison of major key features defining age-related chronic inflammation.

	Age-related inflammation/molecular inflammation	Inflammaging	Senoinflammation
Oxidation	Sirt1, PPAR, FOXOs, SOD, CAT, PTK/PTP	Sirt1, Notch	FOXOs, SOD, CAT, LCK, SRC, PTK/PTP
Inflammation	COX-2, iNOS, TNFα, IL-1,6, AMs	TNFα, IL-6	COX-2, iNOS, TNFα, IL-1,6
Cytokine/Chemokines	IL-7, IL-2RA, CXCL1,2,3, MCP-1, CCL3	TGFβ, IL-8, TNFα	cytokines, chemokines, MMPs, GFs, IGFBPs
Apoptosis	p53, p21, Bax		
Autophagy	mTOR	mTOR	mTOR
Dysregulated metabolism			leptin, adiponectin, anabolism, catabolism
ER stress			IRE, PERK, ATF4,6
Insulin resistance			IRS-Ser-p, Akt
Inflammasome			NLRP3
Reference	6-9, 12-15	10, 11, 54	6-9, 12-15, 23-25, 42, 125

These dangerous effects of senescent cells are largely related to their release of proinflammatory mediators called the senescence-associated (SA) secretome, commonly referred to as SASP, in response to extracellular and intracellular stimuli. Importantly, it is being shown that NF-κB signaling is the major signaling pathway that stimulates the appearance of the SA secretome [76]. The SA secretome includes several families of factors such as cytokines, chemokines, growth factors, and proteases (See Table 1). According to recent studies, cellular senescence is accompanied by a marked increase in the SA secretome of 40-80 factors that participate in intracellular signaling [143]. The most potent SA secretome cytokines are IL-1β, IL-6, and IL-8. These proinflammatory cytokines are increased by DNA damage, replicative exhaustion, and oncogenic stimuli in keratinocytes, melanocytes, monocytes, fibroblasts, and epithelial cells [144-146]. Additional components of the SA secretome are matrix metalloprotease (MMP) family members that are consistently increased in most tissues in which inflammation is present. MMPs are known to regulate inflammation-related activities, including modulation of cytokines and chemokines.

Therefore, SA secretome signaling associated with aging induces a large increase in the secretion of proinflammatory proteins and has emerged as an important additional contributor to chronic inflammation. As shown in Table 2, the SA secretome is upregulated in both senescent cells [144-146] and aged rodent organs [24, 125]. Similar to *in vitro* experiments, and animal and human data, several proinflammatory SA secretome mediators are also upregulated in aged human tissues according to our RNA-seq data comparing normal tissues with tissues from patients with cancer in the TCGA database (unpublished data from our lab).

### Senoinflammation concept as an inclusive schema for age-related chronic inflammation

There are many publications describing the involvement of low-grade inflammation in the aging process and age-related diseases. To explain its diverse implications, various terms and views have been proposed. Included are inflammaging, molecular inflammation, micro-inflammation, pan-inflammation, and gero-inflammation, all of which describe the increased chronic inflammatory activity and proinflammatory mediators associated with aging [147-151], but at present, these age-related chronic inflammation phenomena still remain poorly defined and uncharacterized.

Based on what we now know about chronic inflammation taking place during aging, it seems necessary to formulate a new concept with an expanded scope that can accommodate emerging new data. These new data generated from both within the inflammation domain, and outside of the field, provide diverse views on changes in age-related chronic inflammation that should allow for more integrated approaches to explore the basic mechanisms of aging, as well as for therapeutic intervention.

As shown in Figure 1, the framework of the senoinflammation concept is built on three separate stages that are functionally interdigitated, ranging from the redox-sensitive core transcription factor NF-κB and polarized macrophages, to miRNAs and metabolically linked proinflammatory process, like, ER stress and autophagic activity that have not conventionally been considered part of age-related chronic inflammation. Mechanistically, the senoinflammation concept reveals molecular insights on the complex interaction among diverse transcription factors, inflammatory mediators, and proinflammatory metabolic pathways as being integral, thus, providing a comprehensive chronic inflammation schema for the aging process and age-related diseases.

In our view, the senoinflammation concept proposed here has multiple merits. First, it defines the basic layout for the progressive nature of the inflammation process, resulting in the systemic inflammation seen in chronic inflammation, 2) it provides identifiable proinflammatory mediators and pathways responsible for the sustained inflammation, 3) it reveals the potential interactions among proinflammatory mediators/processes important for propagating the inflammatory condition, and 4) it provides therapeutically targeting selective pro-inflammatory mediators.

### Conclusions

In summary, based on the available findings from biochemical, molecular, and systems analyses, we propose the senoinflammation concept. It provides not only a broader scope, but also creates an intricate network among many inflammatory mediators that can lead to systemic chronic inflammation. When gene regulation is impaired because of constant damage to the genomic DNA by augmented oxidative susceptibility during the aging progresses, several key inflammatory transcription factors, including p53, AP-1, STAT, and NF-κB, that are important in cell survival become over-activated. The resulting aberrant gene regulation in senescent cells leads them into a proinflammatory state, thereby altering systemic chemokine or cytokine activities. The proinflammatory SA secretome imposes further stresses on the intracellular organelles, as well as tissues, organs, and systems, thus influencing metabolic disorders such as insulin resistance. It seems plausible that a vicious cycle takes place between SA secretome induction and metabolic dysregulation, as proposed in the senoinflammation concept, and this may well be the underpinning of the aging process and age-associated diseases.

It is hoped that a better understanding of the molecular mechanisms involved in senoinflammation will provide a basic platform for the identification of potential targets that can suppress age-related chronic inflammation and thereby lead to the development of effective interventions to delay aging and suppress age-associated diseases.
